# Generating and Manipulating High Quality Factors of Fano Resonance in Nanoring Resonator by Stacking a Half Nanoring

**DOI:** 10.1186/s11671-017-2357-5

**Published:** 2017-11-02

**Authors:** Meng Qin, Lingling Wang, Xiang Zhai, Dechao Chen, Shengxuan Xia

**Affiliations:** Key Laboratory for Micro-Nano Optoelectronic Devices of Ministry of Education, School of Physics and Electronics, Hunan University, Changsha, 410082 China

**Keywords:** Optical materials and properties, Nanoparticles, Plasmonic metasurfaces, Fano resonance, Nanoring

## Abstract

We demonstrate the existence of Fano resonance spectral response in a system of nanoscale plasmonic resonant ring stacked by means of a half nanoring. Our proposed scheme exploits the stacked method under normal incidence to excite the subradiant mode. The nanostructure, which utilizes the combination of Fano resonance and polarization-resolved, has a new rotation mode and high tunability, providing a dynamic control of plasmonic spectral response. High-quality resonant line shapes corresponding to the different order modes of Fano structures are readily achieved at near-infrared wavelengths, which is a benefit to the application for nanosensor in highly integrated circuits.

## PACS

73.20.Mf78.67.Bf

## Background

Surface plasmon polaritons (SPPs) have attracted great interest over the past years because of its capability manipulating light-matter interaction in nanoscale dimensions [1–6]. Owing to the fact that the advances in nanofabrication, nano-optical characterization, and improvements in full-field computational electromagnetics, which has led to the emergence of the field of nanoplasmonics, more insight and control has been gained on localized plasmon resonances in metallic nanostructures. In general, plasmon resonances of isolated nanostructures such as disks [7], triangles [8, 9], rods [10, 11], and rings [12, 13] are naturally analyzed. As a fundamental resonant effect, Fano resonances resulting from interference of broad and narrow excitation modes are typically generated in the ring-rod nanostructures [14], plasmonic oligomer clusters [15], nonspherical assemblies [16], graphene-based structures [17], quantum dots [18], etc. In spite of the fact that there are many research efforts, the formation of Fano resonances at specific wavelengths in plasmonic nanostructures is a challenging task because of their complex nature corresponding to the hybridization of the available modes [19–21]. In addition, retardation effects [22, 23] can be varied by the angle of incidence, allowing the existence of dark multipolar modes [24–27], which has recently been exploited in metamaterial context [28–30]. However, this is difficult in systems where higher order modes are excited in the spectral range of interest [31] or when the modes are very complex and spatially extend over a large part of the nanostructure [32]. And the plasmonic nanostructures barely have been studied in a spatially revolved fashion on a subwavelength scale. Information about the spatial distribution of plasmonic nanostructures is crucial to unravel the mechanism that leads to mode generation from plasmonic structures. Furthermore, we can provide a recipe for how one plasmonic element can be efficiently coupled to the other plasmonic component.

In this article, we demonstrate different Fano resonances in a stacked nanostructure composed of individual nanoring and half nanoring. Numerical results from finite-difference time-domain (FDTD) simulations show that the even-order mode of Fano resonance is particularly excited and controlled by stacking method under normal incidence rather than a general method with oblique incidence. Our approach provides new insights into the spectral features of the Fano resonance. The different spectral features associated with multiple Fano resonances each correspond to distinct plasmonic modes. Quite remarkably, the multiple Fano resonances involving the rotation modes, which are based on the different orientation angle of half nanoring, will be achieved. Two high quality factors of Fano resonances with effective dephasing time are simultaneously achieved at spectrum. These results may have potential applications for nanosensor in highly integrated circuits. Furthermore, we show how the geometry of the structure determines the Fano resonance and then how the existent initial modes convert to the different modes for controlling it. This control, which is associated with the properties of the nanostructure, is important very much for practical applications since it provides high design flexibility, remarkable and robust tunability, and excellent performance.

### Methods

**Fig. 1:**
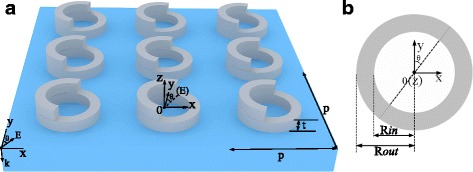
**a** Schematic diagram of the silver nanoring/half nanoring, the geometric parameters are R*in* = 310 nm, R*out* = 400 nm, *t* = 40 nm and *p* = 1000 nm. **b** The top view corresponding to the single cell of nanostructure is arranged right. The orientation angle of the half nanoring shifting from the axle wire (along the *y*-direction) of the concentric system is *θ*

The proposed concentric system composed of a silver (Johnson and Christy) nanoring stacked by a silver half nanoring, as shown schematically in Fig. 1, is investigated to exhibit different radiant modes. Here, the radius of the nanoring/half nanoring inner radius (R*in*) and outer radius of the ring (R*out*) are 310, 400 nm, respectively. For our platform, the amount of the structural handed-helix [33] is determined by the angle *θ*, which is the orientation angle of the half nanoring shifting from the axle wire (along the *y*-direction) of the concentric system. For the structure, the nanoring and half nanoring with the thickness (*t*) are placed on a substrate that the period is *p* and refractive index is set to be 1. The corresponding geometric parameters are given as follows: *t* = 40 nm and *p* = 1000 nm. To perform our numerical calculations by the Lumerical FDTD Solutions, the grid sizes in the *x* and *y* and *z* directions are chosen to be Δ*x* = Δ*y* = Δ*z* = 1 nm [16] and Δ*t* = Δ*x*/2*c*; here, *c* is the velocity of light in a vacuum. The incident plane wave illumination is taken to be along the backward *z*-direction with the polarization along the *y*-direction in the simulations. In addition, the computational domain is truncated by perfectly matched layers (PMLs) in the *z*-direction and the periodical boundary in the *x*- and *y*-directions.

## Results and Discussion

**Fig. 2:**
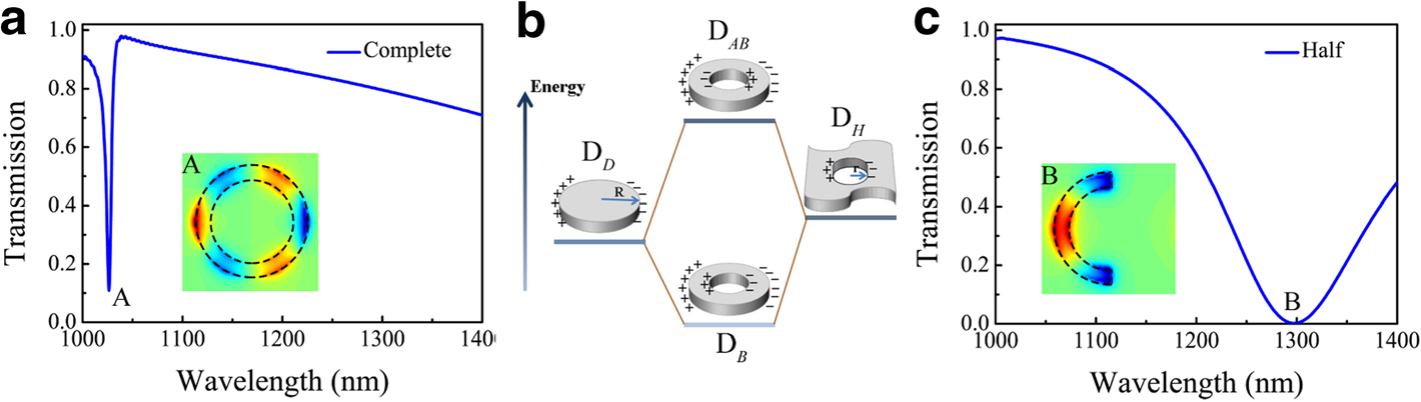
**a** Transmission properties of spectra of complete nanoring alone. The distribution of *z*-component of electric field at the wavelength of 1027 nm denoted by the inset *A.* **b** Mechanism of the plasmonic hybridization between the dipolar modes of the disk (D_*D*_) and anti-dot (hole) (D_*H*_) structures and the energy diagram of the degenerated plasmon bonding (D_*B*_) and antibonding (D_*AB*_) modes. The signs “+” and “−” represent the positive and negative charges, respectively. **c** Transmission spectra of the single half nanoring. The distribution of *z*-component of electric field at the wavelength of 1297 nm denoted by the inset *B*

Figure 2a, c shows the optical properties of the plasmonic nanostructures, which is considered singly. Because the nanostructures exhibited only odd modes of plasmonic resonances at normal incidence [25], the third-order mode of the nanoring at 1027 nm *A* can be excited under the normal illumination with polarization along the *y*-axis, which implies third-order resonance mode of the nanoring is superradiant. In this geometry, the Fano line shape arises from the hybridized coupling between a plasmon resonance of the disk and a plasmon resonance supported by the slice of anti-dot [34, 35], which can be qualitatively described as the dipolar plasmon disk associated with a disk-shaped hole in a metallic film (hole structure) [36], as is shown explicitly in Fig. 2b. From Fig. 2b, we can clearly utilize the plasmon hybridization concept to explain the origin of the third-order Fano resonance where the plasmon modes can be understood as bonding (D_*B*_) or antibanding (D_*AB*_) mode combination of the nanodisk (D_*D*_) and the anti-dot (D_*H*_) plasmon modes. Furthermore, the dipole of single half nanoring first-order mode at 1297 nm *B* is clearly observed, as shown explicitly in Fig. 2c.

**Fig. 3:**
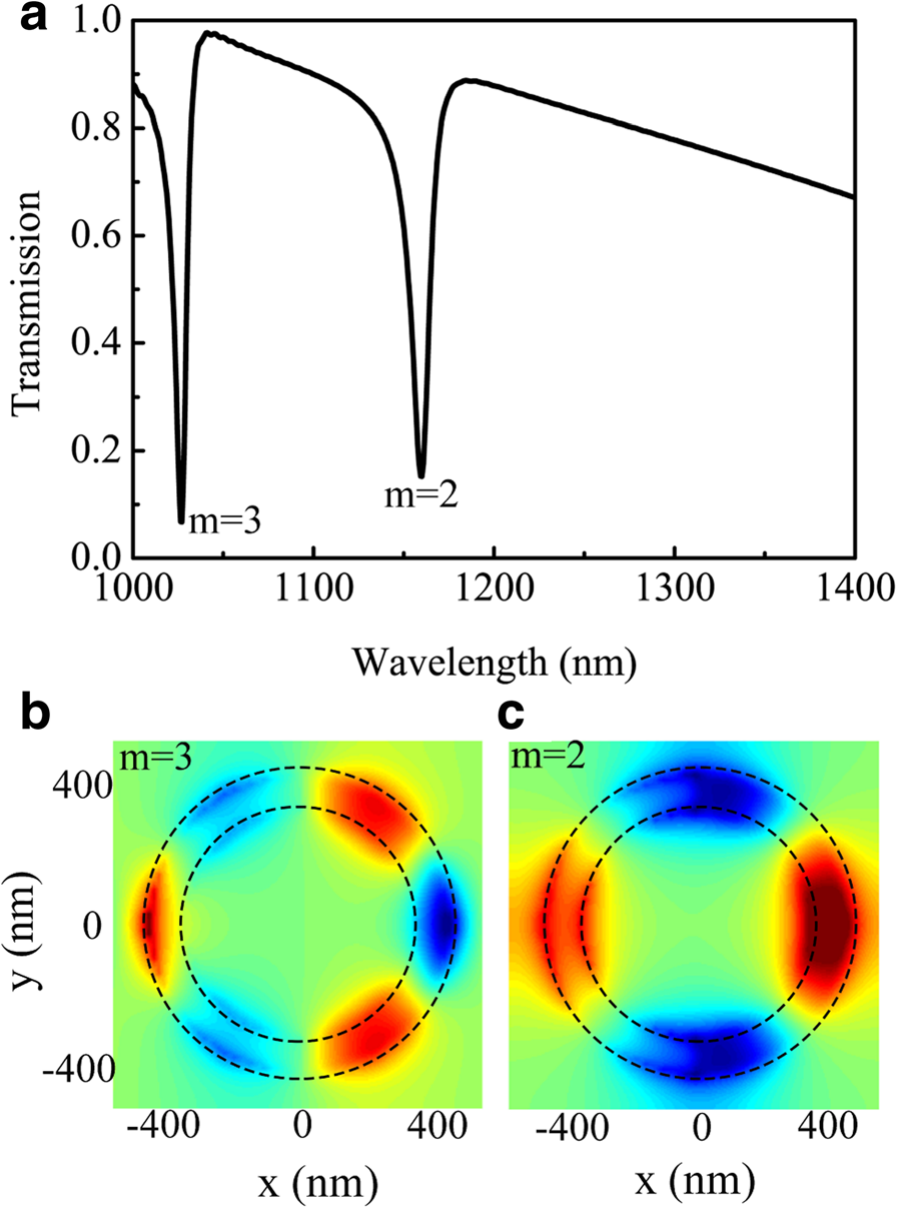
**a** Transmission spectra of the coupled system in Fig. 1 (denoted by black line) through the half silver nanoring coupled with the complete silver nanoring. **b**, **c** Distributions of *z*-component of electric field at wavelengths of 1027 (*m* = 3) and 1160 nm (*m* = 2), respectively

To further elucidate transmission characteristics of the stacked nanostructure, we also calculated that the spectral response of stacked system is a combination of the individual layers modes, as shown in Fig. 3a. In order to completely offset the positive and negative dipole moment, a second-order mode Fano resonance cannot be directly stimulated except for the way of an oblique incidence [22]. Figure 3b shows the third-order mode (*m* = 3) Fano resonance is similar to the previously analyzed case in Fig. 2b. When the half nanoring standing on the nanoring was investigated, the third-order mode (*m* = 3) Fano resonance almost remains invariant. In addition to this, the second-order mode (*m* = 2) Fano resonance efficiency was achieved at a wavelength of 1160 nm, as shown explicitly in Fig. 3c. Comparing the superradiant plasmon resonance modes, we can conclude the Fano resonance arises from stacking influence. And a modification of the circumstance in or around the nanorings affects the resonant mode [10]: its resonance wavelength will change compared with that of the single nanoring or half nanoring. The stacking contact causes a strong blueshift of the fundamental first-order mode, while the geometrical shape of the stacking nanoring/half nanoring still allows for an efficient excitation of higher order modes. These two plasmon resonant first-order modes of the stacking nanoring/half nanoring are blueshifted to 1160 nm, resulting in the existence of second-order mode (*m* = 2) Fano resonances, where the first-order mode of the nanoring at a relatively long wavelength shifts more than the half nanoring’s. We demonstrate that new Fano type resonant modes are excited due to the hybridization between the first-order mode of the nanoring and the half nanoring. Since these two modes can influence each other, it can be ascribed to the compensation of the retardation effect during Fano interference. It is obvious to get that second-order mode (*m* = 2) Fano resonance is governed by the stack of the half nanoring because of the different transmission distributions and propagation characteristics of the nanostructure. As it can be observed, on the one hand, the existence of the half nanoring has little influence on the third-order mode (*m* = 3) Fano resonance, which retains the great characteristics. On the other hand, it shows that the half nanoring has a positive influence on the second-order mode (*m* = 2) Fano resonance. Noticeably, the full width at half-maximum (FWHM) of second-order resonance is 14 nm, showing a quality factor (*Q*-factor) up to 82.8. And we calculated the FWHM of the third-order resonance in the stacked nanostructure to be 9 nm, which is located at 1027 nm effectively with a high quality factor of 114. Two high quality factors in the stacked are achieved by the stacking between its constituting elements, which is larger than 20 [37], 50 [38], and 62 [10]. Furthermore, the dephasing time of the induced resonant mode can crucially influence its properties of the resonances. We calculated the dephasing time of the induced resonant mode via *T*_*r*_ = 2 *ℏ*/*Γ*_*L*_ (*r* = 2, 3) [39–41], where *ℏ* is the reduced Planck’s constant and *Γ*_*L*_ is the homogeneous line width of the Fano resonance. The dephasing time of second-order resonant mode (*m* = 2) *T*_2_ is estimated as 0.10 ps, and the third-order resonant mode (*m* = 3) *T*_3_ is estimated as 0.12 ps. Since the Fano resonances, dephasing times *T*_0_ are believed to be on the order of 10 fs [41] and are thus too short to be reliably resolved with available laser pulses. Both *T*_2_ and *T*_3_ are larger than the general Fano resonances dephasing times *T*_0,_ which can be easily realized.

**Fig. 4:**
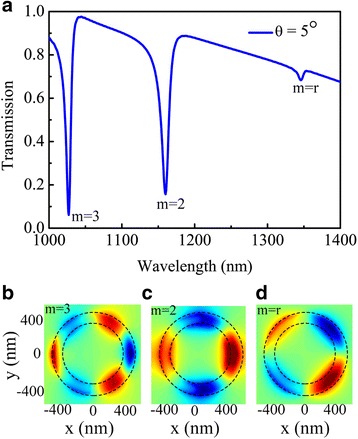
**a** The spectral response of silver nanostructure with a changing angle *θ* = 5° of the half nanoring. Distributions of *z*-component of electric field at wavelengths of 1027 (**b**), 1160 (**c**), and 1346 nm (**d**), respectively

Next, the dependence of Fano resonance on parameters of the system is studied as well. Indeed, as it is the case for the plasmonic resonator, one can select the spectral characteristics of the resonances by changing the handed-helix rotation angle of the half nanoring. When we consider normal incidence with linearly polarized light along the *y*-axis (*θ* = 0°), it can be seen that for *θ* = 0°, only the second- and third-order resonance modes are excited, as shown in Fig. 3a. However, Fig. 4a shows spectra of a slight variation of the handed-helix rotation angle has much more impact on the nanostructures, observing that the 5° rotation of the half nanoring leads to a new mode resonance (named rotation mode *m* = *r*). Clearly, when the half nanoring are placed together with *θ* = 5° rotation, three asymmetric dips exist the spectrum. In order to identify the hybridized modes, we plot the surface charge distributions corresponding to the three dips in the hybridized spectrum, as shown in Fig. 4b–d. The electric field diagram describes the hybridization of the plasmon modes supported by this stacked system. Moreover, one should notice that the third-order mode (*m* = 3) as a superradiant mode under such an excitation almost no change along the *y*-axis of the nanostructure, whereas, the second-order Fano resonance (*m* = 2) is consistent with the mechanism on above, identified as a hybridization first-order mode of half nanoring and the nanoring. Notably, the rotation resonances mode (*m* = *r*) of the nanoring cannot be excited in a single configuration because of the retardation effect. The dip in the wavelength rotation modes (*m* = *r*) is also hybridized between resonance first-order modes of the half nanoring and the nanoring. In the situation of rotation, the Fano resonance shows the same charge distribution with the second-order mode (*m* = 2), but with a structural handed-helix rotation angle, as shown in the charge distribution in Fig. 4d. Based on the second-order mode, the rotation mode is supported a by rotating method and show an asymmetric redshift (shift to long wavelength). The revolving half nanoring has double function that one is used as a half nanoring to generate the second-order mode and the other is served as a revolving half nanoring to excite the rotation mode. Note that the resonance of the dip in the spectrum can strengthen or vanish, resulting in the flexible modulation in integrated circuits.

**Fig. 5:**
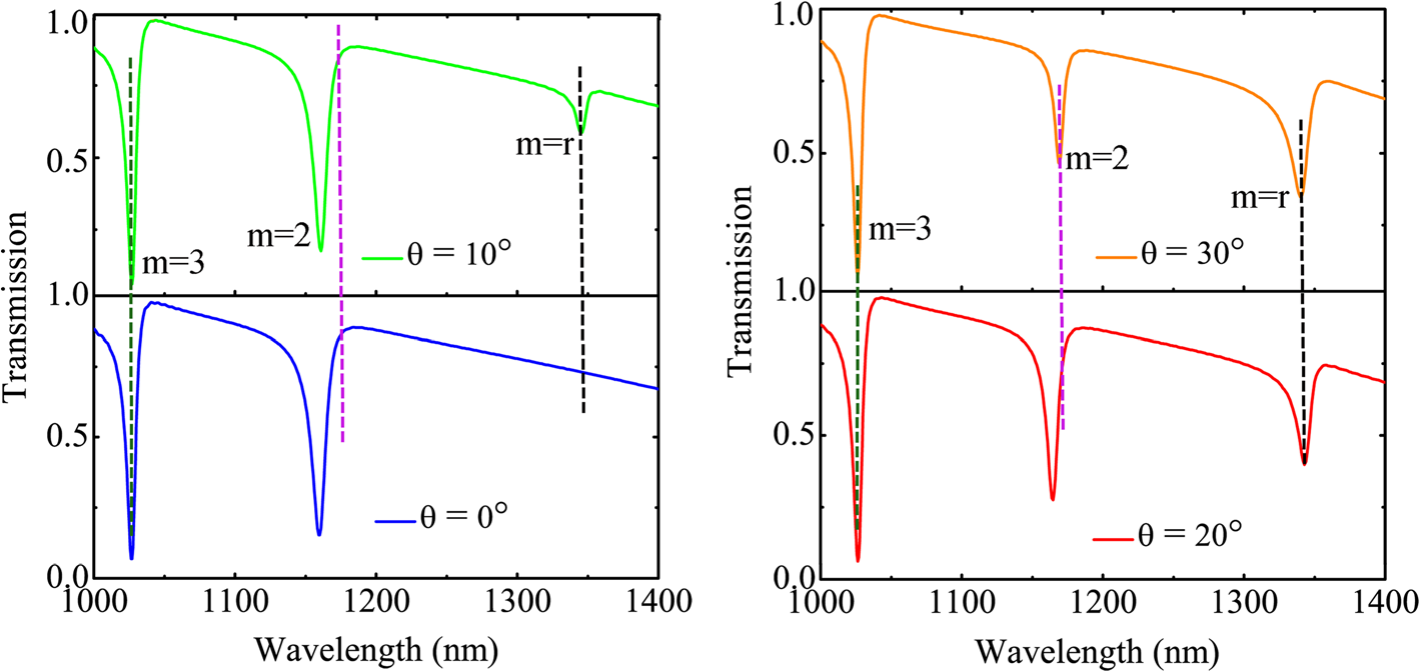
The blue, green, red, and origin line present the simulation transmission spectra for various right-hand rotation angle *θ* = 0°, 10°, 20°, and 30°, respectively, with the other parameters same as Fig. 1

Figure 5 shows spectra of nanostructure with the same diameters but with a changing handed helix angle of the half nanoring deviating from the direction of the electric field polarization. The angle difference leads to the variation of the rotation resonant mode (*m* = *r*), which is in agreement with the above analysis of the modes. When the angle difference becomes very large, such as in the case from *θ* = 0° to *θ* = 30°, the line shape of the hybridized spectrum becomes more distinctive. It can be seen that the mode (*m* = *r*) is not dominant enough since the half nanoring has a little rotation moment due to its little angle size. And the revolving resonant mode becomes apparent increasing with the angle. Thus, the whole structure exhibits three modes. Additionally, the second-order mode (*m* = 2) becomes decreased since the net moment along the *y*-axis is small, which results in weak interference insufficient for a distinctive Fano profile in second-order mode (*m* = 2). As the angle of the half nanoring becomes larger, the resonant difference becomes obvious, so that the overlap of the two modes is prominent, making the asymmetric Fano profile (*m = r*) more distinctive.

It is interesting to see that for nanostructure composed of a nanoring with the same length but a half nanoring, a distinctive (actually much sharper) second-order mode Fano resonance can also be excited, which can stimulate two high-quality Fano at the same time, contributing to development of integrated circuits. This further demonstrates that the special shape of the nanoring makes different from those in other nanoparticle systems. The reason about the particular behavior of plasmon hybridization is that for the half nanoring where their ends are relatively docking nanoring, where the strong influence will induce the even-mode of the nanostructure. But as the angle of the half nanoring are varied, the rotating mode (*m* = *r*) are excited, which subsequently produces three Fano resonance profiles. Of course, when the half nanoring shift to another direction from the *y*-direction of the concentric system (in the case of *θ* = 0°, − 10°, − 20°, − 30°), the phenomenon of the nanostructure is same as in Fig. 5. We can draw the same conclusions that a slight variation of the rotation angle has much more impact on the nanoring resonant modes. There is the new mode resonance (rotation mode *m* = *r*) consistent with the descriptions before.

## Conclusions

In summary, a novel silver plasmonic nanostructure that combines mode resonances into a hybrid system, which consists of nanoring stacked by a half nanoring, supporting a Fano resonance in the near-infrared range of the spectrum, has been analyzed and investigated. The nanostructure exhibits high tunability and robust control of its spectral features with only a few structural handed-helix rotation parameters. The analysis of the electric field distribution revealed that the different modes can be excited for specific frequencies. Otherwise, multiple Fano resonances are achieved by rotating the angle of half nanoring and then the mechanisms are significantly perturbed. The stack of a half nanoring creates a path for realizing different Fano resonance modes in the plasmonic resonant system. In addition, the Fano line shapes are of high quality factor that can be easily applied for nanosensor in highly integrated circuits.

## Abbreviations

FDTD: Finite-difference time-domain
FWHM: Full width at half-maximum
PMLs: Perfectly matched layers
*Q*-factor: Quality factor
SPPs: Surface plasmon polaritons
